# Tris(1*H*-benzimidazol-2-ylmeth­yl)amine methanol tris­olvate

**DOI:** 10.1107/S2414314620002813

**Published:** 2020-03-05

**Authors:** Bertin Anzaldo-Olivares, Maribel Arroyo, Armando Ramírez-Monroy, Sylvain Bernès

**Affiliations:** a Centro de Química del Instituto de Ciencias, Benemérita Universidad Autónoma de Puebla, Ciudad Universitaria, 72570 Puebla, Pue., Mexico; b Instituto de Física, Benemérita Universidad Autónoma de Puebla, Av. San Claudio y 18 Sur, 72570 Puebla, Pue., Mexico; Dublin City University, Ireland

**Keywords:** crystal structure, tripodal N-donor ligand, methanol solvate, hydrogen bonds, mol­ecular conformation

## Abstract

The structure of the tertiary amine tris­(1*H*-benzimidazol-2-ylmeth­yl)amine (C_24_H_21_N_7_, abbreviated ntb) has been previously reported twice as solvates, namely the monohydrate and the aceto­nitrile–methanol–water (1/0.5/1.5) solvate, both with the tripodal conformation formed *via* multiple hydrogen bonds. Now, we report the tri­methanol adduct, ntb·3CH_3_OH, where the amine has the stair conformation featuring one benzimidazole group oriented in the opposite direction from the other two. The asymmetric unit contains one-half amine, completed through the mirror plane *m* in space group *Pmn*2_1_ to form the ntb mol­ecule, with the H atom for each imidazole moiety equally disordered between both N sites available in the imidazole ring. The asymmetric unit also contains one and a half methanol mol­ecules, one being placed in general position with the hy­droxy H atom disordered over two sites with occupancy ratio 1:1, while the other lies on the *m* mirror plane, and has thus its hy­droxy H atom disordered by symmetry. As in the previously reported solvates, all imine and amine groups of the ntb mol­ecules and the methanol mol­ecules are involved in N—H⋯O and O—H⋯N hydrogen bonds. In the title compound, however, the involved H atom is systematically a disordered H atom provided by an imidazole group or a methanol mol­ecule.

## Abstract 2

In the title solvate, the tertiary amine adopts a stair-like conformation, with all imine and amine groups forming N—H⋯O and O—H⋯N hydrogen bonds with the lattice methanol mol­ecules.

## Graphical abstract

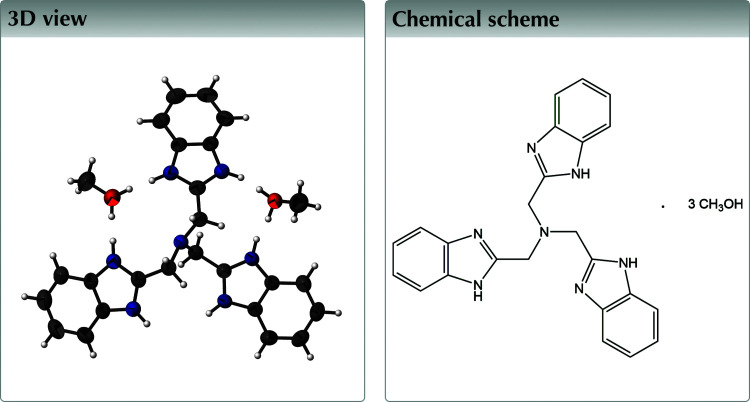

## Structure description

The tertiary amine tris­(1*H*-benzimidazol-2-ylmeth­yl)amine (abbreviated ntb in the literature) is a mol­ecule that can act as a tetra­dentate ligand through its three imine N atoms and the central amine one, exhibiting thus a versatile and rich coordination chemistry, where the mol­ecule adopts a tripodal coordination mode to the metal (*e.g*. Nakata *et al.*, 1997 ▸; Kwak *et al.*, 1999 ▸; Rheingold & Hammes, 2015 ▸). In addition, it is a potential hydrogen-bond donor through its three NH amine groups (Su *et al.*, 2000 ▸). Similar tripodal behaviour has been found in ntb solvent adducts such as the monohydrate, C_24_H_21_N_7_·H_2_O, and the aceto­nitrile–methanol–water (1/0.5/1.5) solvate, C_24_H_21_N_7_·C_2_H_3_N·0.5CH_4_O·1.5H_2_O. In both cases, ntb forms two N—H⋯O and one O—H⋯N hydrogen bonds with a solvent water mol­ecule (Zhang *et al.*, 2005 ▸). In addition, the imine N atoms of the benzimidazolyl arms can be protonated with HNO_3_, and the resulting trication then adopts a stair arrangement, where the N atoms of the benzimidazolium fragments inter­act with anions NO_3_^−^ through N—H⋯O hydrogen bonds (Cui, 2011 ▸).

**Figure 1:**
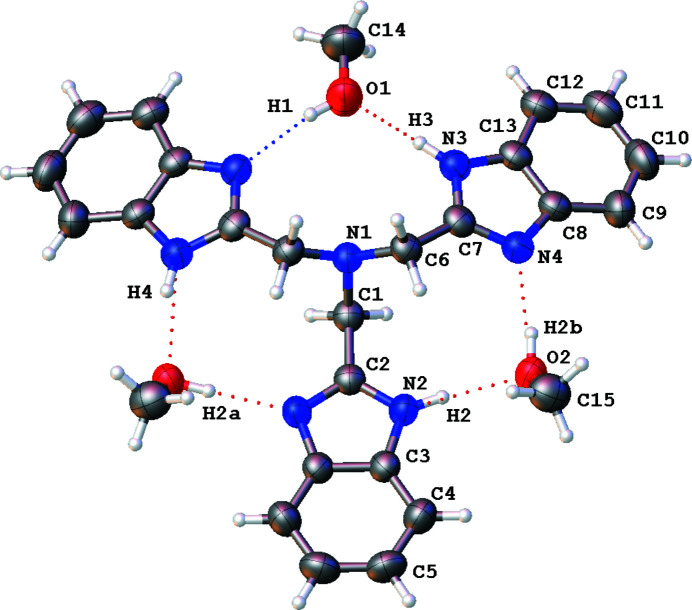
Mol­ecular structure of the title compound, with displacement ellipsoids for non-H atoms at the 50% probability level. For all disordered H atoms, a single site was retained, in order to emphasize the O—H⋯N and N—H⋯O hydrogen bonds (dotted lines). Non-labelled atoms are generated by the symmetry operation 1 − *x*, *y*, *z*.

**Figure 2:**
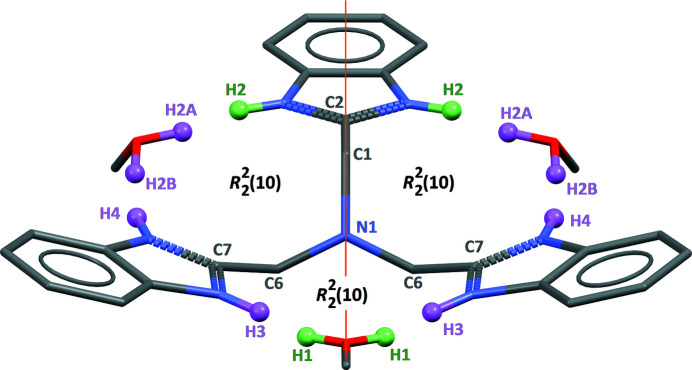
View of the title compound rotated by *ca* 90° in comparison with Fig. 1 ▸, showing the stair-like conformation for ntb. All H atoms bonded to heteroatoms are shown. Green H atoms are disordered by symmetry, while purple H atoms are positionally disordered, all having the same half occupancy. The orange line is the trace of the *m* mirror normal to the *a* axis.

The present report deals with a new ntb solvate. When this amine is crystallized from methanol solution, the solvate ntb·3CH_3_OH is obtained, in space group *Pmn*2_1_. The ntb mol­ecule has one benzimidazolyl arm placed over the mirror *m* plane (N2/C1⋯C5 group), while the other crystallographically independent arm (N3/N4/C6⋯C13 group) is placed in general position (Fig. 1 ▸). As a consequence, instead of the common tripodal mol­ecular geometry (approximate point group: *C*_3v_), the ntb mol­ecule adopts a stair-like conformation (point group: *C*_s_), with one benzimidazole group oriented in the opposite direction from the other two (Fig. 2 ▸). The imidazol H atom of the arm bis­ected by the mirror plane, H2, is disordered by symmetry so that the π-bond of this imidazole ring is delocalized over C2 N2 and C2 N2^*i*^ bonds [symmetry code: (i) 1 − *x*, *y*, *z*], with a bond length of 1.342 (3) Å. The same kind of delocalization is observed in the other ntb arm, with the imidazolic H atom equally disordered over N3 and N4, and identical bond lengths C7 N3 and C7 N4 of 1.338 (4) and 1.335 (4) Å. In other words, amine and imine sites in ntb are indistinguishable (Fig. 2 ▸). As clearly observed in Fig. 1 ▸, the methanol molecules of crystallization are recognized by the ntb mol­ecule in a similar way as in the previously described ionic compound ntb·3HNO_3_ (Cui, 2011 ▸). The methanol mol­ecules are sandwiched between two ntb arms, in order to form three O—H⋯N and three N—H⋯O hydrogen bonds (Table 1 ▸), with O⋯N separations spanning a short range, from 2.722 (4) to 2.767 (3) Å. Given that all imidazolic H atoms are disordered, the same holds for hy­droxy H atoms: the first methanol mol­ecule C14—O1 lies in the mirror *m* plane, with its H atom disordered by symmetry (H1); the second methanol mol­ecule, C15—O2, placed in general position, has its hy­droxy H atom disordered over two sites, H2*A* and H2*B*, with half occupancy (Fig. 2 ▸). With this arrangement, any physically unreasonable H⋯H contact is avoided, despite all heteroatoms, except N1, being involved in efficient hydrogen bonds. The complete adduct ntb·3CH_3_OH thus features three similar ring motifs 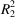 (10) sharing the central N1 atom (Fig. 2 ▸).

The supra­molecular structure is further extended by one-dimensional stacks of ntb mol­ecules in the [011] direction. The stair conformation adopted by ntb allows rather close π–π inter­actions between benzimidazole rings along the stacks: mean plane separations for neighbouring rings along a stack are 3.782 (2) and 3.454 (2) Å for the N2/C2–C5 and N3/N4/C7–C13 benzimidazole rings, respectively.

## Synthesis and crystallization

The title compound was prepared through the solid-solid condensation reaction of nitrilo­tri­acetic acid (1 g, 5.2 mmol) and *o*-phenyl­enedi­amine (1.7 g, 15.7 mmol), both reactants contained in a round-bottom flask provided with a septum connected to the outside through a needle, which was maintained at 463–473 K in a sand bath for 1 h. As the heating progressed, a dark-brown solution formed, which solidified when the temperature returned to 298 K. Then, 0.7 g of activated carbon and 20 ml of methanol were added and the mixture refluxed for 2 h. The still hot mixture was filtered in a Büchner funnel, giving an orange filtrate. The product was isolated by successive recrystallizations from methanol in 30% yield, and single crystals grew by slow evaporation at 298 K as colourless needles with a melting point of 543–547 K. IR (KBr): 3391, 3177, 1624, 1535, 1437, 1273, 1119, 737 cm^−1^.

## Refinement

Crystal data, data collection and structure refinement details are summarized in Table 2 ▸. The crystal emulates a tetra­gonal system (*a* ≃ *b*), however, extinctions and structure refinement are consistent with space group *Pmn*2_1_. The imidazole ring in a general position (N3/H3/N4/H4/C7/C8/C13) was restrained to be flat, within a standard deviation of 0.1 Å^3^ (Sheldrick, 2015*b* ▸).

## Supplementary Material

Crystal structure: contains datablock(s) I. DOI: 10.1107/S2414314620002813/gg4004sup1.cif

Structure factors: contains datablock(s) I. DOI: 10.1107/S2414314620002813/gg4004Isup2.hkl

Supporting information file. DOI: 10.1107/S2414314620002813/gg4004Isup3.cml

CCDC reference: 1987232

Additional supporting information:  crystallographic information; 3D view; checkCIF report

## Figures and Tables

**Table 1:** Hydrogen-bond geometry (Å, °)

*D*—H⋯*A*	*D*—H	H⋯*A*	*D*⋯*A*	*D*—H⋯*A*
O1—H1⋯N3^i^	0.84 (3)	1.95 (4)	2.762 (3)	164 (10)
O2—H2*A*⋯N2^i^	0.86 (3)	1.91 (3)	2.767 (3)	174 (9)
O2—H2*B*⋯N4	0.86 (3)	1.87 (3)	2.722 (4)	171 (8)
N2—H2⋯O2^i^	0.88 (3)	1.92 (3)	2.767 (3)	161 (7)
N3—H3⋯O1	0.88 (3)	1.88 (3)	2.762 (3)	176 (6)
N4—H4⋯O2	0.88 (3)	1.90 (3)	2.722 (4)	156 (3)

Symmetry code: (i) 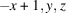 .

**Table 2:** Experimental details

Crystal data	
Chemical formula	C_24_H_21_N_7_·3CH_4_O
*M*_r_	503.60
Crystal system, space group	Orthorhombic, *P* *m* *n*2_1_
Temperature (K)	293
*a*, *b*, *c* (Å)	16.7781 (17), 16.3902 (18), 4.7894 (5)
*V* (Å^3^)	1317.1 (2)
*Z*	2
Radiation type	Mo *K*α
μ (mm^−1^)	0.09
Crystal size (mm)	0.77 × 0.17 × 0.10

Data collection	
Diffractometer	Agilent Xcalibur, Atlas, Gemini
Absorption correction	Analytical (*CrysAlis PRO*; Agilent, 2012 ▸)
*T*_min_, *T*_max_	0.975, 0.995
No. of measured, independent and observed [*I* > 2σ(*I*)] reflections	8328, 2938, 1903
*R*_int_	0.047
(sin θ/λ)_max_ (Å^−1^)	0.641

Refinement	
*R*[*F* ^2^ > 2σ(*F* ^2^)], *wR*(*F* ^2^), *S*	0.048, 0.101, 1.03
No. of reflections	2938
No. of parameters	196
No. of restraints	11
H-atom treatment	H atoms treated by a mixture of independent and constrained refinement
Δρ_max_, Δρ_min_ (e Å^−3^)	0.14, −0.14

Computer programs: *CrysAlis PRO* and *CrysAlis RED* (Agilent, 2012 ▸), *SHELXT2018/2* (Sheldrick, 2015*a* ▸), *SHELXL2018/3* (Sheldrick, 2015*b* ▸), *OLEX2* (Dolomanov *et al.*, 2009 ▸), *Mercury* (Macrae *et al.*, 2020 ▸) and *publCIF* (Westrip, 2010 ▸).

## full crystallographic data

### Crystal data

**Table:**

C_24_H_21_N_7_·3CH_4_O	*D*_x_ = 1.270 Mg m^−^^3^
*M**_r_* = 503.60	Melting point: 543 K
Orthorhombic, *P**m**n*2_1_	Mo *K*α radiation, λ = 0.71073 Å
*a* = 16.7781 (17) Å	Cell parameters from 1461 reflections
*b* = 16.3902 (18) Å	θ = 3.8–23.1°
*c* = 4.7894 (5) Å	µ = 0.09 mm^−^^1^
*V* = 1317.1 (2) Å^3^	*T* = 293 K
*Z* = 2	Needle, colourless
*F*(000) = 536	0.77 × 0.17 × 0.10 mm

### Data collection

**Table:**

Agilent Xcalibur, Atlas, Gemini diffractometer	2938 independent reflections
Radiation source: fine-focus sealed X-ray tube, Enhance (Mo) X-ray Source	1903 reflections with *I* > 2σ(*I*)
Graphite monochromator	*R*_int_ = 0.047
Detector resolution: 10.5564 pixels mm^-1^	θ_max_ = 27.1°, θ_min_ = 3.5°
ω scans	*h* = −21→21
Absorption correction: analytical (CrysAlis PRO; Agilent, 2012)	*k* = −20→20
*T*_min_ = 0.975, *T*_max_ = 0.995	*l* = −6→6
8328 measured reflections	

### Refinement

**Table:**

Refinement on *F*^2^	Secondary atom site location: difference Fourier map
Least-squares matrix: full	Hydrogen site location: mixed
*R*[*F*^2^ > 2σ(*F*^2^)] = 0.048	H atoms treated by a mixture of independent and constrained refinement
*wR*(*F*^2^) = 0.101	*w* = 1/[σ^2^(*F*_o_^2^) + (0.0376*P*)^2^] where *P* = (*F*_o_^2^ + 2*F*_c_^2^)/3
*S* = 1.03	(Δ/σ)_max_ < 0.001
2938 reflections	Δρ_max_ = 0.14 e Å^−^^3^
196 parameters	Δρ_min_ = −0.14 e Å^−^^3^
11 restraints	Extinction correction: SHELXL-2018/3 (Sheldrick 2015b), Fc^*^=kFc[1+0.001xFc^2^λ^3^/sin(2θ)]^-1/4^
0 constraints	Extinction coefficient: 0.011 (2)
Primary atom site location: dual	

### Special details

**Table:**

Refinement. All C-bonded H atoms were placed in calculated positions and refined as riding atoms, while H atoms bonded to N (H2, H3, H4) and O sites (H1, H2A, H2B) were found in difference maps, and refined with free coordinates and occupancy fixed to 1/2. Corresponding bond lengths were restrained to O—H = 0.85 (2) and N—H = 0.90 (2) Å.

### Fractional atomic coordinates and isotropic or equivalent isotropic displacement parameters (Å^2^)

**Table:**

	*x*	*y*	*z*	*U*_iso_*/*U*_eq_	Occ. (<1)
N1	0.500000	0.7530 (2)	0.4852 (7)	0.0411 (8)	
N2	0.43371 (14)	0.56374 (16)	0.6509 (6)	0.0483 (6)	
H2	0.3840 (19)	0.578 (4)	0.616 (15)	0.058*	0.5
N3	0.63616 (15)	0.85800 (15)	0.2727 (6)	0.0481 (7)	
H3	0.593 (3)	0.886 (3)	0.231 (13)	0.058*	0.5
N4	0.71198 (16)	0.76093 (15)	0.4609 (6)	0.0517 (7)	
H4	0.725 (4)	0.7189 (17)	0.565 (8)	0.062*	0.5
C1	0.500000	0.6713 (3)	0.3595 (8)	0.0456 (11)	
H1A	0.546531	0.667019	0.240219	0.055*	0.5
H1B	0.453469	0.667019	0.240219	0.055*	0.5
C2	0.500000	0.5995 (3)	0.5526 (8)	0.0441 (11)	
C3	0.45864 (17)	0.5001 (2)	0.8202 (7)	0.0467 (8)	
C4	0.4157 (2)	0.4429 (2)	0.9728 (8)	0.0620 (9)	
H4A	0.360238	0.442753	0.973673	0.074*	
C5	0.4586 (2)	0.3865 (2)	1.1222 (9)	0.0674 (10)	
H5	0.431646	0.347357	1.226065	0.081*	
C6	0.57325 (16)	0.77133 (19)	0.6402 (7)	0.0461 (7)	
H6A	0.588978	0.723567	0.746291	0.055*	
H6B	0.562722	0.815084	0.771494	0.055*	
C7	0.64012 (19)	0.79584 (19)	0.4532 (7)	0.0471 (8)	
C8	0.75915 (19)	0.80341 (19)	0.2720 (7)	0.0495 (8)	
C9	0.8387 (2)	0.7945 (2)	0.1972 (8)	0.0657 (10)	
H9	0.870759	0.754618	0.277911	0.079*	
C10	0.8681 (2)	0.8473 (3)	−0.0016 (9)	0.0733 (11)	
H10	0.920992	0.842674	−0.057055	0.088*	
C11	0.8205 (3)	0.9071 (2)	−0.1211 (9)	0.0712 (11)	
H11	0.842493	0.941876	−0.253804	0.085*	
C12	0.7416 (2)	0.9164 (2)	−0.0482 (8)	0.0590 (9)	
H12	0.709832	0.956296	−0.129831	0.071*	
C13	0.71147 (18)	0.86342 (18)	0.1529 (7)	0.0470 (8)	
C14	0.500000	1.0001 (4)	−0.0870 (13)	0.0875 (19)	
H14A	0.477295	1.049629	−0.015783	0.131*	0.5
H14B	0.468974	0.981180	−0.242542	0.131*	0.5
H14C	0.553731	1.010105	−0.146720	0.131*	0.5
O1	0.500000	0.9415 (3)	0.1199 (10)	0.0815 (11)	
H1	0.458 (3)	0.923 (6)	0.19 (2)	0.122*	0.5
C15	0.7479 (2)	0.5979 (2)	0.9575 (8)	0.0747 (11)	
H15A	0.768010	0.543691	0.985412	0.112*	
H15B	0.702487	0.606585	1.075551	0.112*	
H15C	0.788684	0.636824	1.003105	0.112*	
O2	0.72533 (13)	0.60776 (15)	0.6755 (6)	0.0605 (7)	
H2A	0.676 (2)	0.595 (5)	0.68 (2)	0.091*	0.5
H2B	0.727 (5)	0.657 (2)	0.611 (17)	0.091*	0.5

### Atomic displacement parameters (Å^2^)

**Table:**

	*U*^11^	*U*^22^	*U*^33^	*U*^12^	*U*^13^	*U*^23^
N1	0.043 (2)	0.043 (2)	0.0379 (19)	0.000	0.000	−0.0032 (16)
N2	0.0407 (14)	0.0496 (16)	0.0546 (14)	−0.0032 (13)	−0.0037 (15)	0.0040 (14)
N3	0.0464 (16)	0.0483 (16)	0.0496 (15)	−0.0033 (12)	−0.0017 (14)	0.0020 (15)
N4	0.0483 (16)	0.0488 (16)	0.0580 (16)	−0.0015 (14)	−0.0008 (15)	0.0056 (14)
C1	0.046 (2)	0.050 (3)	0.041 (2)	0.000	0.000	−0.004 (2)
C2	0.047 (3)	0.044 (3)	0.041 (3)	0.000	0.000	−0.0046 (19)
C3	0.0454 (17)	0.0455 (18)	0.0492 (16)	−0.0028 (14)	−0.0037 (15)	−0.0035 (15)
C4	0.052 (2)	0.062 (2)	0.072 (2)	−0.0085 (18)	0.001 (2)	0.006 (2)
C5	0.078 (2)	0.053 (2)	0.072 (2)	−0.0092 (17)	0.004 (2)	0.013 (2)
C6	0.0480 (18)	0.0465 (18)	0.0436 (16)	−0.0053 (14)	−0.0038 (17)	−0.0014 (16)
C7	0.050 (2)	0.0470 (19)	0.0445 (17)	−0.0089 (16)	−0.0031 (16)	−0.0069 (17)
C8	0.052 (2)	0.0430 (18)	0.0535 (19)	−0.0070 (16)	−0.0004 (17)	−0.0004 (17)
C9	0.048 (2)	0.067 (2)	0.082 (3)	−0.0003 (18)	0.006 (2)	0.001 (2)
C10	0.056 (2)	0.079 (3)	0.085 (3)	−0.013 (2)	0.017 (2)	−0.003 (2)
C11	0.076 (3)	0.065 (3)	0.073 (3)	−0.016 (2)	0.013 (2)	0.002 (2)
C12	0.065 (2)	0.051 (2)	0.061 (2)	−0.0083 (17)	0.003 (2)	0.0023 (19)
C13	0.0523 (19)	0.0416 (18)	0.0472 (17)	−0.0087 (15)	−0.0005 (18)	−0.0047 (16)
C14	0.088 (4)	0.076 (4)	0.099 (5)	0.000	0.000	0.030 (4)
O1	0.061 (2)	0.085 (3)	0.099 (3)	0.000	0.000	0.039 (2)
C15	0.084 (3)	0.072 (3)	0.068 (3)	−0.006 (2)	−0.009 (2)	0.004 (2)
O2	0.0478 (13)	0.0639 (16)	0.0698 (17)	0.0046 (13)	0.0019 (15)	0.0169 (14)

### Geometric parameters (Å, º)

**Table:**

N1—C6^i^	1.467 (3)	C6—H6B	0.9700
N1—C6	1.467 (3)	C8—C9	1.390 (4)
N1—C1	1.468 (5)	C8—C13	1.390 (4)
N2—C2	1.342 (3)	C9—C10	1.377 (5)
N2—C3	1.386 (4)	C9—H9	0.9300
N2—H2	0.88 (3)	C10—C11	1.388 (5)
N3—C7	1.338 (4)	C10—H10	0.9300
N3—C13	1.391 (4)	C11—C12	1.378 (5)
N3—H3	0.88 (3)	C11—H11	0.9300
N4—C7	1.335 (4)	C12—C13	1.391 (4)
N4—C8	1.389 (4)	C12—H12	0.9300
N4—H4	0.88 (3)	C14—O1	1.379 (6)
C1—C2	1.496 (6)	C14—H14A	0.9600
C1—H1A	0.9700	C14—H14B	0.9600
C1—H1B	0.9700	C14—H14C	0.9600
C3—C3^i^	1.388 (6)	O1—H1	0.84 (3)
C3—C4	1.391 (4)	O1—H1^i^	0.84 (3)
C4—C5	1.373 (5)	C15—O2	1.412 (4)
C4—H4A	0.9300	C15—H15A	0.9600
C5—C5^i^	1.388 (7)	C15—H15B	0.9600
C5—H5	0.9300	C15—H15C	0.9600
C6—C7	1.491 (4)	O2—H2A	0.86 (3)
C6—H6A	0.9700	O2—H2B	0.86 (3)
			
C6^i^—N1—C6	113.8 (3)	N3—C7—C6	123.8 (3)
C6^i^—N1—C1	113.3 (2)	N4—C8—C9	131.5 (3)
C6—N1—C1	113.3 (2)	N4—C8—C13	107.1 (3)
C2—N2—C3	106.5 (3)	C9—C8—C13	121.4 (3)
C2—N2—H2	127 (4)	C10—C9—C8	117.1 (4)
C3—N2—H2	127 (4)	C10—C9—H9	121.5
C7—N3—C13	105.7 (3)	C8—C9—H9	121.5
C7—N3—H3	126 (4)	C9—C10—C11	121.6 (4)
C13—N3—H3	129 (4)	C9—C10—H10	119.2
C7—N4—C8	106.4 (3)	C11—C10—H10	119.2
C7—N4—H4	125 (4)	C12—C11—C10	121.7 (4)
C8—N4—H4	128 (4)	C12—C11—H11	119.1
N1—C1—C2	117.6 (3)	C10—C11—H11	119.1
N1—C1—H1A	107.9	C11—C12—C13	117.1 (3)
C2—C1—H1A	107.9	C11—C12—H12	121.4
N1—C1—H1B	107.9	C13—C12—H12	121.4
C2—C1—H1B	107.9	C8—C13—N3	108.0 (3)
H1A—C1—H1B	107.2	C8—C13—C12	121.1 (3)
N2—C2—N2^i^	111.9 (4)	N3—C13—C12	130.9 (3)
N2—C2—C1	124.06 (19)	O1—C14—H14A	109.5
N2^i^—C2—C1	124.06 (19)	O1—C14—H14B	109.5
N2—C3—C3^i^	107.56 (15)	H14A—C14—H14B	109.5
N2—C3—C4	131.2 (3)	O1—C14—H14C	109.5
C3^i^—C3—C4	121.2 (2)	H14A—C14—H14C	109.5
C5—C4—C3	117.1 (3)	H14B—C14—H14C	109.5
C5—C4—H4A	121.5	C14—O1—H1	122 (7)
C3—C4—H4A	121.5	C14—O1—H1^i^	122 (7)
C4—C5—C5^i^	121.7 (2)	H1—O1—H1^i^	116 (10)
C4—C5—H5	119.2	O2—C15—H15A	109.5
C5^i^—C5—H5	119.2	O2—C15—H15B	109.5
N1—C6—C7	112.4 (3)	H15A—C15—H15B	109.5
N1—C6—H6A	109.1	O2—C15—H15C	109.5
C7—C6—H6A	109.1	H15A—C15—H15C	109.5
N1—C6—H6B	109.1	H15B—C15—H15C	109.5
C7—C6—H6B	109.1	C15—O2—H2A	102 (7)
H6A—C6—H6B	107.8	C15—O2—H2B	116 (6)
N4—C7—N3	112.9 (3)	H2A—O2—H2B	106 (7)
N4—C7—C6	123.2 (3)		
			
C6^i^—N1—C1—C2	−65.7 (2)	N1—C6—C7—N4	−127.8 (3)
C6—N1—C1—C2	65.8 (2)	N1—C6—C7—N3	56.5 (4)
C3—N2—C2—N2^i^	−0.9 (5)	C7—N4—C8—C9	179.1 (4)
C3—N2—C2—C1	179.8 (3)	C7—N4—C8—C13	−0.7 (3)
N1—C1—C2—N2	89.6 (4)	N4—C8—C9—C10	179.8 (3)
N1—C1—C2—N2^i^	−89.6 (4)	C13—C8—C9—C10	−0.5 (5)
C2—N2—C3—C3^i^	0.5 (3)	C8—C9—C10—C11	0.4 (6)
C2—N2—C3—C4	−179.5 (3)	C9—C10—C11—C12	−0.4 (6)
N2—C3—C4—C5	179.9 (4)	C10—C11—C12—C13	0.5 (6)
C3^i^—C3—C4—C5	−0.2 (4)	N4—C8—C13—N3	0.9 (3)
C3—C4—C5—C5^i^	0.2 (4)	C9—C8—C13—N3	−178.9 (3)
C6^i^—N1—C6—C7	−149.0 (2)	N4—C8—C13—C12	−179.6 (3)
C1—N1—C6—C7	79.7 (4)	C9—C8—C13—C12	0.6 (5)
C8—N4—C7—N3	0.2 (4)	C7—N3—C13—C8	−0.8 (3)
C8—N4—C7—C6	−176.0 (3)	C7—N3—C13—C12	179.8 (3)
C13—N3—C7—N4	0.4 (3)	C11—C12—C13—C8	−0.6 (5)
C13—N3—C7—C6	176.5 (3)	C11—C12—C13—N3	178.8 (3)

Symmetry code: (i) −*x*+1, *y*, *z*.

### Hydrogen-bond geometry (Å, º)

**Table:**

*D*—H···*A*	*D*—H	H···*A*	*D*···*A*	*D*—H···*A*
O1—H1···N3^i^	0.84 (3)	1.95 (4)	2.762 (3)	164 (10)
O2—H2*A*···N2^i^	0.86 (3)	1.91 (3)	2.767 (3)	174 (9)
O2—H2*B*···N4	0.86 (3)	1.87 (3)	2.722 (4)	171 (8)
N2—H2···O2^i^	0.88 (3)	1.92 (3)	2.767 (3)	161 (7)
N3—H3···O1	0.88 (3)	1.88 (3)	2.762 (3)	176 (6)
N4—H4···O2	0.88 (3)	1.90 (3)	2.722 (4)	156 (3)

Symmetry code: (i) −*x*+1, *y*, *z*.
